# Detection of nontoxic BoNT/A levels in post-facial Botox injection breastmilk

**DOI:** 10.3389/fdsfr.2024.1480515

**Published:** 2025-01-06

**Authors:** Helene Gu, Zhenyu Xu, Renata Koviazina, Pengcheng Tan, Changcheng Zheng, Ferdinand Kappes, Domna G. Kotsifaki, Fangrong Shen, Anastasia Tsigkou

**Affiliations:** ^1^ Reproductive Oncology Lab, Division of Natural and Applied Sciences, Duke Kunshan University, Kunshan, Jiangsu, China; ^2^ Photonics Lab, Division of Natural and Applied Sciences, Duke Kunshan University, Kunshan, Jiangsu, China; ^3^ Optical Characterization Lab, Division of Natural and Applied Sciences, Duke Kunshan University, Kunshan, Jiangsu, China; ^4^ Division of Natural and Applied Sciences, Duke Kunshan University, Kunshan, Jiangsu, China; ^5^ First Affiliated Hospital, Soochow University Department of Obstetrics and Gynecology, Suzhou, Jiangsu, China

**Keywords:** breastfeeding, botulinum neurotoxin type A, ELISA, Western blot, confocal micro-Raman spectroscopy, mass spectrometry (LC-MS)

## Abstract

The use of cosmetic Botox (BoNT/A) has become increasingly prevalent among women, even during the post-pregnancy breastfeeding period. However, there is currently a limited understanding of the extent Botox enters breastmilk and its potential effect on the breastfeeding infant. In this study, breastmilk samples were acquired from five women aged between 28 and 45. Three sample sets ranged from 1 h to 1 year after facial Botox treatments (64 U), whereas the remaining two were from women who never received Botox. BoNT/A concentrations in samples were detected using standard Enzyme-Linked Immunosorbent Assay (ELISA), unreduced and reduced Western Blotting, confocal micro-Raman Spectroscopy, and Mass Spectrometry (LC-MS). From ELISA, the greatest breastmilk BoNT/A concentration was found from woman 1, 4 days after Botox injection (167 pg/mL). Levels were highest overall in the first week (82.45–167 pg/mL) and around 2 months (132.725 pg/mL) after injection. No clear indication of BoNT/A was detected in Mass Spectrometry (LC-MS), Western Blotting and confocal micro-Raman Spectroscopy, but Western blot and confocal micro-Raman Spectroscopy show promise of development into future means of detection. From our study, the amount of BoNT/A in breastmilk peaks around 4 days (167 pg/mL) and at 2 months (132.725 pg/mL) after facial injection. Even over a year after injection, BoNT/A can be detected. However, all quantities of BoNT/A detected (between 34.4 pg/mL and 167 pg/mL) are likely to be safe for infants.

## Graphical Abstract

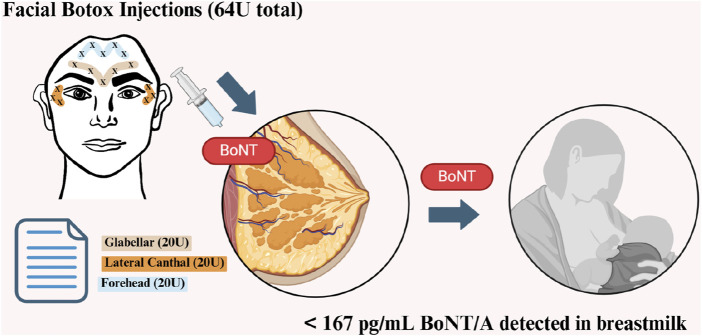

## Highlights

• BoNT/A was detected in the breastmilk of 3 women after facial Botox injections of 64 U.
• Toxin levels peaked in the first week and at around 2 months after injection.
• All detected levels (up to 167 pg/mL) were significantly below the lethal dose for newborns.

## 1 Introduction

In today’s social media oriented culture, caring about one’s appearance is becoming the new normal. Consequently, there has been a rapid growth of cosmetic procedures, with the most popular, Botox, having developed into a seven billion dollar industry (Hood and Wilson, 2001). These accessible treatments are also seen in pregnant women. During pregnancy, women’s bodies change with expanding bellies, growing breasts, swelling ankles/wrists, and stretch marks developing in the skin as they grow a child. After pregnancy, the immense pressure to “bounce back” evokes body dissatisfaction in some mothers (Gavin et al., 2005; Sweeney and Fingerhut, 2013; Riesco-González et al., 2022). Without strong social support, body insecurities and celebrity posts of a more glamorous motherhood can be significant motivators for some women to seek timely Botox treatments, even during the early lactation period (Williams, 2013). However, it is unknown whether this represents a risk to the newborn.

Although injected Botox is regarded as safe, BoNT/A has a history of being a deadly toxin when ingested orally (Ting and Freiman, 2004). Given that an oral dose as small as 1 μ g/kg may be lethal, a lethal dose of Botox for a 3.5 kg baby would be 3.5 μ g (Arnon et al., 2001). In addition to lower body weight, newborns’ less developed immune systems may also make botulinum neurotoxin particularly dangerous, causing diminished facial expressions, loss of muscle tone, difficulty feeding, and general weakness (Bakheit et al., 1997).

In clinical treatments for pregnant women, Botox doses have varied from 1.25 to 300 U (Brandt et al., 2009; Morgan et al., 2006). Cosmetic Botox doses are lower, with a standard of 20 U for dermatology (Satriyasa, 2019). One U, i. e., mouse unit, refers to the lethal dose that kills 50% of mice (Ting and Freiman, 2004). For humans, a lethal dose of Botox by injection is around 3000 U (Ting and Freiman, 2004). Therefore, Botox treatments have almost unanimously been found to be safe and free from long lasting side effects in clinical trials (Brandt et al., 2009). Due to a high molecular weight and neurospecificity to its intramuscular targets, BoNT/A, the active ingredient of Botox, is not likely to spread far systemically or enter breastmilk (Hilderbrand et al., 1961). However, many other drugs have been detected in breastmilk previously (Sachs and Drugs, 2013; Julsgaard et al., 2023).

A recent study on Botox was conducted in four patients receiving facial injections. In two of the patients (receiving 54 U and 92 U, respectively), no BoNT/A was detected in breastmilk (Hudson et al., 2024). In the other two women, between 85.24 and 746.82 pg/mL were detected 0–5 days post-injection (Hudson et al., 2024). In the LactMed database, other case reports related to Botulinum toxin and breastmilk are sparse and are related to maternal botulism from food, not Botox injections (Sachs and Drugs, 2013). These cases are few in number, sample size, and time points measured.

Breastmilk, produced in the alveoli of breasts, derives its nutrients from the circulatory system, thus being prone to contamination by contents of the mother’s bloodstream (Sachs and Drugs, 2013). Despite potential harms, the benefits of breastmilk are wide-spanning and well-studied. Breastfeeding benefits both the mother and newborn by reducing obesity, diabetes, hypertension, cardiovascular disease, and certain cancers (Binns et al., 2016; Neves et al., 2021). For the newborn, breastmilk from the first 2 days after birth provides high amounts of antibodies and immune cells. Afterwards, it continues to provide important proteins, sugars, vitamins, minerals, hormones, and growth factors (Bhora et al., 1995; Ahmed et al., 2004). For the mother, breastfeeding can reduce stress, fatigue, and depression (Figueiredo et al., 2014). Therefore, the World Health Organization (WHO) recommends mothers to initiate breastfeeding within the first hours of life and exclusively breastfeed for at least 6 months (Tucker and O’Malley, 2022; World Health Organization WHO, 2023). However, mothers with certain diseases or maternal medications proven to pose risks to babies are an exception and are advised not to breastfeed (Centers for Disease Control and Prevention CDC, 2023).

With the currently limited number of studies published, the Food Drug Administration (FDA) warns against women using Botox during pregnancy and breastfeeding (Medication Guide BOTOX Cosmetic for Injection, 2011). Our study aims to inform this recommendation by quantifying BoNT/A levels in breastmilk post-injection via standard, i. e., ELISA, and non-standard i.e., Western Blotting, confocal micro-Raman spectroscopy, and mass spectrometry techniques.

## 2 Materials and methods

### 2.1 Study design and participants

The study participants were three lactating women between the ages of 35 and 45 who underwent their most recent Botox treatment more than a year ago. Additionally, two breastfeeding women aged 28 to 32 who have never received Botox treatment served as controls (Table 1). Women received BoNT/A injections (BOTOX^®^ Cosmetic onabotulinumtoxinA, United States) treatment for glabellar lines, lateral canthal lines, and forehead lines in accordance with the FDA Botox medication guide (BOTOX, 1989). They received 20 U, 24 U, and 20 U at the 3 sites respectively, totaling 64 U per woman. All women were instructed to express around 100 mL of breastmilk per breast for 30 min using a manual pump. Notably, it cannot be excluded that not all milk in the breast was collected by this method. Around 200 mL of each sample was collected in separate labeled sterile bags (MEDELA,ZG) before their latest treatment and 1, 6, 24, 36, 48, and 72 h after treatment. All women received no further Botox treatments for the entire duration of the study. Samples were immediately frozen in participants’ home freezers at −20 °C and transported to the laboratory at day 4 using dry ice. Samples were stored −80 °C until analysis. In addition, further samples from some women were collected in the first 2 weeks at 5, 6, 7, 10, and 11 days, and at 2 months, 4 months, and 5 months (Table 1) following the same above procedure, under which botulinum neurotoxin can be stable for years (Malizio et al., 2000).

**TABLE 1:** Summary of participant data and samples collected from 3 women who have received facial Botox injections and 2 women who have never received Botox.All women had weights between 48–65 kg and heights between 160–175 cm.

Woman	Injection type	Age	Race	Disease state	Breastmilk samples collected
1	Facial (64 U)	35–45	Caucasian	None	1 h, 6 h, 24 h, 36 h, 48 h, 72 h, 4 d, 5 d, 6 d, 11 d, 2 m, 4 m, 5 m, 1+ year
2	Facial (64 U)	35–45	Caucasian	None	6 h, 24 h, 36 h, 48 h, 72 h, 5 d, 7 d, 1+ year
3	Facial (64 U)	35–45	Caucasian	None	24 h, 36 h, 48 h, 4 d, 5 d, 6 d, 11 d, 1+ year
4	Never received Botox	28	Asian	None	Normal breastmilk random sample
5	Never received Botox	32	Asian	None	Normal breastmilk random sample

### 2.2 Sample preparation and dialysis

Breastmilk sample aliquots were centrifuged at 1,500 × g at 4 °C for 15 min and the skim milk fraction was collected through a disposable 5 mL needle syringe. The skim milk sample was centrifuged again at 3,000 × g at 4 °C for 30 min to remove remaining fat globules and cell debris. Samples were stored as 1 mL aliquots at −80 °C for further analysis.

Dialysis of breastmilk was performed overnight at 4 °C in a cold room with 50 kDa MWCO dialysis membranes (Shyuanye,CN) and deionized (DI) water as the solute. Anti-BoNT/A (AbCam, United Kingdom) antibodies were bound and then covalently cross-linked to the bead-immobilized protein A (Beyotime, CN). To produce a stable immunomatrix, the Seize X Immunoprecipitation Kit (Pierce, United States) was used as described by the manufacturer. The beads were centrifuged and washed three times with 500 μ L “Gentle Elution buffer” and two times with 500 μ L “Gentle Binding” buffer (Pierce, United States). Spin Cup columns with functionalized beads were wrapped with parafilm and stored at 4 °C until used. Breastmilk proteins were subjected to affinity chromatography with immobilized antibodies and were rotated at 8 rpm at 4 °C overnight, then centrifuged at 1,700 × g for 10 min, and washed three times with 500 μ L of PBS buffer.

### 2.3 Enzyme-linked immunosorbent assay (ELISA)

Breastmilk BoNT/A concentrations were measured in duplicates by a commercial ELISA kit for Human Botulinum Toxin Type A (BTX-ELISA, China). ELISA was performed on samples spanning from 6 h to 5 months following the manufacturer’s instructions and as outlined (Table 2).

**TABLE 2:** Breastmilk samples loaded in ELISA plate wells. Each sample was loaded in duplicate. The first row consists of the standards provided by the manufacturer to produce the standard curve.

	1	2	3	4	5	6	7	8	9	10	11	12
A	ST 0		ST 1		ST 2		ST 3		ST 4		ST 5	
	(0 pg/mL)		(5 pg/mL)		(10 pg/mL)		(20 pg/mL)		(40 pg/mL)		(50 pg/mL)	
B	Woman 1		Woman 1		Woman 1		Woman 1		Woman 1		Woman 1	
	6 h		24 h		36 h		48 h		72 h		4 days	
C	Woman 1		Woman 1		Woman 1		Woman 1		Woman 1		Woman 1	
	5 days		7 days		11 days		2 months		4 months		5 months	
D	Woman 4		Woman 5		Woman 2		Woman 3		Woman 3		Woman 3	
	no Botox		no Botox		24 h		36 h		48 h		72 h	

#### 2.3.1 ELISA plate analysis

The ELISA plate was read by a Varioskan LUX microplate reader (Thermo Fisher Scientific, Inc., United States). Values of each pair of duplicates were averaged. BoNT/A concentration versus absorbance of the standard curve was plotted following the manufacturer’s manual. The absorbance of standard 1 (0 pg/mL) was subtracted from all other absorbance values. From these adjusted average absorbances, concentrations of the breastmilk samples were determined. See the Supplementary Appendix for full ELISA methods.

### 2.4 Polyacrylamide gel electrophoresis (PAGE) and Western Blot

The next method applied was Western Blotting. Western Blotting included non-reducing and reducing methods in which BoNT/A bands would be expected at different kDAs. The inactive BoNT/A protein has a molecular weight of 150 kDa, consisting of a light chain (50 kDa) and heavy chain (100 kDa) connected by a disulfide bond (Rossetto et al., 2006).

The detection used a primary antibody specific to the 1,280 - $1292^{th}$ amino acid residues of the heavy chain of the BoNT/A protein. To confirm the presence of sulfide bonds, we compared between reducing and non-reducing PAGE, anticipating the identification of a product approximately 150 kDa (non-reducing PAGE) or 100 kDa (reducing PAGE) (Dressler and Benecke, 2007).

See the Supplementary Appendix for full Western Blotting methods.

#### 2.4.1 Non-reducing PAGE

Samples from 24, 36, and 48 h were subjected to polyacrylamide gel electrophoresis using non-reducing conditions. In this procedure, the toxin shows only the protein band formed by units of molecular weight at 150 kDa.

#### 2.4.2 Reducing PAGE

Samples across a longer span of time were subjected to reducing electrophoresis. In reducing PAGE, the toxin is resolved into its heavy (molecular weight = 100 kDa) and light (molecular weight = 50 kDa) subunits due to breakage of disulfide bonds (Grenda et al., 2021).

### 2.5 Confocal micro-Raman Spectroscopy

Next we were interested in investigating the BoNT/A concentration in breastmilk with a non-standard technique, i.e., confocal micro-Raman Spectroscopy.

Raman spectra for samples of woman 1 at 48 h, 72 h, and 1+ year, samples from woman 2 at 6 h and 1 year, and negative control samples from women 4 and 5 were collected at room temperature using a confocal micro-Raman Spectrometer (LabRAM HR Evolution, HORIBA Scientific, JP). See the Supplementary Appendix for the full methods.

### 2.6 Liquid chromatography - mass spectrometry (LC-MS)

Liquid Chromatography - Mass Spectrometry (LC-MS) was used to analyze unprocessed breastmilk samples of 1 mL taken at 6 and 48 h post-Botox administration. Mass spectrometry was outsourced to the company PTM Bio Hangzhou China. See the Supplementary Appendix for the full methods.

## 3 Results

**FIGURE 1:**
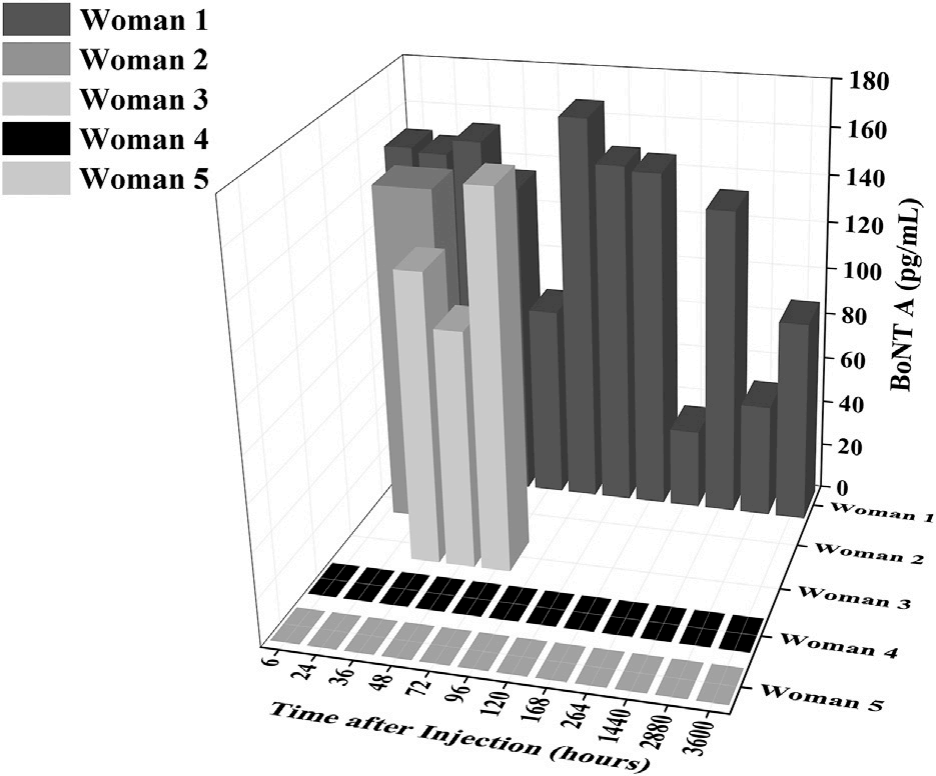
Concentrations of BoNT/A in ELISA samples as a function of time and as a function of breastmilk samples. The level of BoNT/A in breastmilk generally decreases over time after the first week (6–168 h) but has a distinct peak again at 2 months (1,440 h).

Using a commercially available ELISA kit, concentrations of BoNT/A ranging from 0 to 167 pg/mL were detected in all breastmilk samples of women that received Botox treatment, while no BoNT/A was detected in the control group (Figure 1). The highest concentration observed was in the sample from woman 1, 4 days after the injection (167 pg/mL). This is in line with the Hudson et al. observations that found peaks at 3 and 5 days, respectively, in two women (Hudson et al., 2024). We observed that after the first week, circulating BoNT/A concentrations decrease to a low concentration of 24.4 pg/mL at 11 days, before another peak of 132.725 pg/mL at 2 months (Figure 1).

### 3.1 Western blot results

In the negative control lysate, samples from woman 4 and woman 5, who have never had received treatment of BoNT/A showed the absence of 150 kDa marker for BoNT/A. The Western blots of samples from women post-Botox revealed the presence of fluctuating 150 kDa and 100 kDa (See Supplementary Appendix).

### 3.2 Raman spectroscopy results

In Raman Spectroscopy, several characteristic peaks of breastmilk and BoNT/A were detected (See Supplementary Appendix). Peaks at 918 $cm^{-1}$ and 1,384 $cm^{-1}$ were observed in samples 48 h (woman 1) and 6 h (woman 2) suggesting the presence of BoNT/A (Zhang et al., 2021). These were missing in the in Raman spectra of women 4 and 5’s breastmilk.

### 3.3 Mass spectrometry results

In the 6 h sample, 22,666 peptides were detected, consisting of 22,097 unique peptides and 3,191 identified proteins. In the 48 h sample, 22,333 peptides were detected, with 21,688 unique peptides and 3,140 identified proteins (See Supplementary Appendix). BoNT/A was not clearly detected among these peptides.

## 4 Discussion

Botox has been extensively used in clinical treatments for conditions such as excessive sweating (hyperhidrosis), muscle stiffness (spasticity), enlarged facial muscles (facial muscular hypertrophy), muscle spasms, migraines and cosmetic treatments (Young and Young, 2021). As more women delay pregnancies and seek to continue breastfeeding beyond the WHO-recommended 2 years, and with others experiencing migraines or desiring post-pregnancy improvements, there is insufficient information available regarding the safety of using BoNT/A during breastfeeding for infants. Due to the currently limited information on the detection of BoNT/A in breastmilk, we tested the above methods to provide a better sense of the risk versus benefit assessment.

To some extent, almost all medications transfer from blood to breastmilk (Sachs and Drugs, 2013). The transfer of drugs into breastmilk is influenced by protein binding, lipid solubility, and ionization (Wanat, 2020). With regards to the pharmacokinetics of Botox in the mother, Botox inhibits acetylcholine secretion in neurons of the peripheral nervous system, entering the nerve terminal almost immediately after injection (Bakheit et al., 1997; Carruthers et al., 2005; Schantz and Johnson, 1997). The binding and internalization of Botox have a half-time of 10 min at body temperature, i. e., 36 °C (Black and Dolly, 1986). Within 12 h after injection, it is either absorbed by nerve terminals or released from the muscles (Ahmed et al., 2004; Brooks, 1954). The half-life for Botox in the circulatory system is several days before it reaches target organs (Al-Saleem et al., 2008). Due to its high neurospecificity, it undergoes little biotransformation and does not accumulate in circulating cells, remaining largely in the free state (Sachs and Drugs, 2013). This is in line with our results with peaks in the first week.

With the quantitative BoNT/A breastmilk concentrations determined by ELISA, our concentrations were lower than the 2024 Hudson et al. study, by about 2 to 10-fold (Hudson et al., 2024). The recent study from Hudson et al. found that 8 of the 16 samples had varying levels of botulinum toxin ranging from 85.24 to 746.82 pg/mL, while 8 samples had no toxin detected (Hudson et al., 2024). The variation in outcomes might be influenced by the ELISA kits which detect different epitopes. Enzyme linked immunoassays (ELISA) vary in sensitivity and specificity mainly due to different types of both detection and capture antibodies (Rød et al., 2017). For instance, commercial ELISA kits tested different values of total corticosterone in the same serum samples (Rød et al., 2017). Metabolic variances in the breakdown of Botox could also have played a role. The body’s uptake, circulation, and excretion of BoNT/A is complicated, and metabolism can differ based on genetics or lifestyle choices. In particular, BoNT/A uptake to nerve endings increases with activity and temperature, reducing initial Botox concentrations but increasing later concentrations circulation (Hallett, 2015).

However, the duration of our study continued longer, with samples up to 5 months after Botox injection. The effects of Botox injection decrease after 2 months when it is fully metabolized, and it is generally at 3–4 months when effects completely wear off and a new injection is needed (Brandt et al., 2009). Which corresponds to the second peak 2 months after injection. Because this study design anticipated the peaks to be in the first days, the samples collected later in the study were more sparse (at 2 months, 4 months, 5 months). Therefore, further work could be done with more samples around 2–4 months collected and tested to more accurately determine this second peak.

One additional unanticipated result was exactly how long traces of BoNT/A can remain in the body. Due to prior treatment of the lactating women over 1 year before this study, the original negative “pre Botox” controls we used in Western blots were from women 1, 2, and 3. Because these women had all received Botox procedures for a time longer than 1 year ago, this led to inconclusive results and being unable to find differentiating molecular weight bands on the gel. However, upon acquiring breastmilk samples from women 4 and 5, who had never had Botox before in their lifetime, bands at 150 kDa were not observed. This indicates that even after over a year since a facial injection, detectable amounts of BoNT/A may still be present in circulation.

Our study and previous ones have had a limited sample size and cover only facial Botox injection procedures. Future studies can be done to further elucidate how Botox metabolism changes with different ages, race, physical characteristics, injections, and lifestyles. Maternal concentrations of BoNT/A were not measured, but future work could measure serum levels in mothers. Our study was also limited in that adverse effects on the breastfed infants were not measured. Beyond lethal dosage, the No Observed Effect Level (NOEL) is also of interest. Because oral BoNT/A intake is highly dangerous with no medicinal use, there are no reports of an oral NOEL. However, 2 studies of mothers with Botulism breastfeeding have reported no adverse effects in the infants (Middaugh, 1978; Geiger, 1938). Future work to determine urine, stool, or blood concentrations of BoNT/A in infants and monitor adverse effects of Botulism would be further directions. However, given that the highest value was 167 pg/mL, this is less than 1 in 20,000ths of the lethal dose 3.5 μ g. Hence, for lethal concentrations, 21 L would be required.

Based on an average intake of 670 mL of breastmilk/day, a breastfed infant would ingest 0.112 μ g of Botox toxin in 24 h (Rios-Leyvraz and Yao, 2023). Even using the high end of the breastmilk/day output (840 mL) as an upper limit to ingestion, this results in 0.140 μ g (Anderson and Valdés, 2015). Therefore, it is unlikely that the risks of Botox levels in breastmilk are high enough to outweigh the benefits of breastmilk for the infant and mother.

Based on our knowledge, this study is the first to examine breastmilk from lactating women for presence of BoNT/A using Western blot, confocal micro-Raman Spectroscopy, and Mass Spectrometry. While concentrations were too low for Mass Spectrometry to detect, Western Blotting and confocal micro-Raman Spectroscopy showed promise of future development for detecting Botox (See Supplementary Appendix). In terms of methods, ELISA was the most effective for our purposes, as it had a clear standard curve and straightforward interpretation. The BTX-ELISA kit also had high specificity, with detection and quantification limits in pg/mL based on the standard solutions provided. Together with the results from Hudson et al., this study supports the conclusion that facial BoNT/A injections does not require the interruption of breastfeeding (Hudson et al., 2024).

## Data Availability

The raw data supporting the conclusions of this article will be made available by the authors upon request.
