# Supplementary material for: Detection of nontoxic BoNT/A levels in post-facial Botox injection breastmilk
Source: Front Drug Saf Regul. 2025 Jan 6;4:1480515. doi: 10.3389/fdsfr.2024.1480515 (PMC12443105; doi:10.3389/fdsfr.2024.1480515)
Supplement: Supplementary file 1 [file DataSheet1.pdf]

Highlights

Supplementary Methods, Results, and Discussion: Detection of Nontoxic BoNT/A Levels in Post-Facial Botox Injection Breast-milk

Helene Gu, Zhenyu Xu, Renata Koviazina, Pengcheng Tan, Changcheng Zheng, Ferdinand Kappes, Domna G. Kotsifaki, Fangrong Shen, Anastasia Tsigkou

- Commercial Enzyme Linked Immunosorbent (ELISA) is our current recommendation for BoNT/A detection in breastmilk
- Western Blotting and Raman Spectroscopy techniques show promise for development
- Mass Spectrometry (LC-MS) is likely not specific enough for the levels of BoNT/A present

Supplementary Methods, Results, and Discussion: Detection of Nontoxic BoNT/A Levels in Post-Facial Botox Injection Breastmilk

Helene Gu[a], Zhenyu Xu[b], Renata Koviazina[a], Pengcheng Tan[a], Changcheng Zheng[d], Ferdinand Kappes[c], Domna G. Kotsifaki[b], Fangrong Shen[e], Anastasia Tsigkou[a]

[a]*Reproductive Oncology Lab, Division of Natural and Applied Sciences, Duke Kunshan University, 8 Duke Ave, Kunshan, Jiangsu Province, 215316, China*
[b]*Photonics Lab, Division of Natural and Applied Sciences, Duke Kunshan University, 8 Duke Ave, Kunshan, Jiangsu Province, 215316, China*
[c]*Division of Natural and Applied Sciences, Duke Kunshan University, 8 Duke Ave, Kunshan, Jiangsu Province, 215316, China*
[d]*Optical Characterization Lab, Division of Natural and Applied Sciences, Duke Kunshan University, 8 Duke Ave, Kunshan, Jiangsu Province, 215316, China*
[e]*First Affiliated Hospital, Soochow University Department of Obstetrics and Gynecology, 296 Shizi St, Cang Lang Qu, Suzhou, Jiangsu Province, 215005, China*

1. Supplementary Methods

1.1. ELISA Details

The Enzyme Linked Immunosorbent Assay (ELISA) was analyzed according to the manufacturer's manual as follows. 50 µL of each standard and 5x diluted sample was loaded in duplicate then incubated at 37°C for 60 minutes with 100 µL of HRP-conjugate reagent. The wells were washed 5 times and aspirated until dry. 50 µL of chromogen solution A and chromogen solution B were added to each well and incubated for 15 minutes at 37°C away from light. Finally, 50 µL of reaction stopping solution were added and wells were gently tapped. A Varioskan LUX microplate reader (Thermo Fisher Scientific, Inc., USA) was used to read the Optical Density (O.D.) at 450 nm. The average reading of the duplicates was taken.

*Corresponding author: Anastasia Tsigkou: anastasia.tsigkou@dukekunshan.edu.cn

1.1.1. ELISA Calculations

First, the value of the standard (0 pg/mL) was subtracted from all other cells. Next, a linear concentration curve was generated from the standards, with the 0 pg/mL standard having an absorbance of 0. Using this equation, the O.D. readings of the other wells were converted into concentrations of BoNT/A. Due to the 5x dilution, these values were multiplied by 5 to obtain the concentrations of the original breastmilk samples.

1.2. Polyacrylamide Gel Electrophoresis (PAGE) and Western Blot

1.2.1. Non-reducing PAGE

In the first experiment, samples from 24, 36, and 48 hours were subjected to polyacrylamide gel electrophoresis using non-reducing conditions. In this procedure, the toxin shows only the protein band formed by units of molecular weight at 150 kDa.

1.2.2. Reducing PAGE

In the second experiment, samples across a longer span of time were subjected to reducing electrophoresis. In reducing PAGE, the toxin is resolved into its heavy (molecular weight = 100 kDa) and light (molecular weight = 50 kDa) subunits due to breakage of disulfide bonds [1].

1.2.3. Western Blotting

For all Western Blots, total protein concentration was quantified using BCA Protein assay (Thermo Fisher Scientific, Inc.,USA). A total of 30 μg of proteins in breastmilk samples were separated on 8 or 10% Tris-Glycine gels (Titan Scientific, CN). Before loading, proteins were denatured using 3 μL 5× SDS (Sodium Dodecyl Sulfate) and heated at 95°C for 5 mins. For reduced PAGE, 3 μL of beta-mercaptoethanol was also added before heating in order to break the disulfide bridge connecting the heavy (100 kDa) and light (50 kDa) chains of BoNT/A. This was not added to the unreduced PAGE.

After (SDS) - electrophoresis, proteins were transferred onto a polyvinyldiene fluoride (PVDF) membrane in a Mini Trans-Blot cell (Bio-Rad, CA, USA) at a constant current of 315 mA on ice for 90 min. The membrane was incubated for one hour at room temperature with a blocking solution of PBS/BSA 5%+NaN_3, then washed for 5 min in Tris-buffered saline (TBS: 150 mM NaCl, 10 mM Tris-HCl (pH 7.4)), to remove the excess BSA (Bovine Serum Albumin protein). After blocking, mouse anti-clostridium botulinum

toxin A [B364M] (ab252737) primary antibody diluted in 2% BSA/PBS was added to the membrane and incubated overnight at 4°C on a shaker. The membrane was then washed once with Tris-buffered saline-Tween (TBST: 150 (AbCam,UK) mM NaCl, 10 mM Tris-HCl, and 0.1% Tween 20) for 15 min, followed by three washes with TBS (15 min each). The membrane was then incubated at room temperature for 1 hour with a clean blot secondary antibody (1 : 3000 in TBS) (Sigma-Aldrich Inc., CN) to suppress signals from antibodies naturally present in human breastmilk [2]. The membrane was then washed once in TBST for 15 min and 4 times in TBST for 5 min and left in AP buffer (100 mM Tris-HCl (pH 9.5), 100 mM NaCl, and 5 mM $MgCl_2$) for 5 min. The bands were detected with a ChemiDoc Imaging system (Bio-Rad) and normalized to the expression of GAPDH, using rabbit polyclonal antibody (bs-10900R) (BiOss, CN) by semi-quantitative analysis.

Applicable controls including loading control, negative control lysate, and no-primary-antibody control were conducted in accordance with the antibody producer's protocol.

1.3. Confocal micro-Raman Spectroscopy

Raman spectra were collected at room temperature using a confocal micro-Raman Spectrometer (LabRAM HR Evolution, HORIBA Scientific, JP). A diode laser beam at 532 nm with power at the sample plane of 3.2 mW was used to provide better Raman efficiency compared to longer wavelengths. The laser beam was focused using a high numerical aperture (NA = 1.25) oil immersion objective lens (Plan N 100×, Olympus) onto the sample to maximise the Raman signal. The spectrometer has a spectral resolution of 1.0 cm^{-1} and a spectral range of 100 - 4000 cm^{-1}. Each spectrum was averaged from three scans with an integration time of 15 s. Using adhesive microscope spacers with a thickness of 120 μm, a microwell was formed on the microscope glass slide, and a spectrum occurred in the 6 μL sample solution under a glass cover slide. The Raman spectra were analyzed using LabSpec6 software to identify the peaks. The analysis included baseline correction before peak position determination. Then the Savitzky–Golay filter was used for spectra smoothing by a 3rd-degree polynomial function.

1.4. Liquid Chromatography - Mass Spectrometry (LC-MS)

Liquid Chromatography - Mass Spectrometry (LC-MS) was used to analyze unprocessed breastmilk samples of 1 mL taken at 6 and 48 hours post-Botox administration. Liquid Chromatography was conducted using a Van-

quish Neo UPLC system (Thermo Fisher Scientific, Inc.,USA). The Mass spectrometry was analyzed using an Orbitrap Astral with a nano-electrospray ion source. Analysis methods including Gene Ontology (GO), Domain Annotation, KEGG, Subcellular Localization, COG/KOG, Reactome, WikiPathways, Hallmark Signature Gene sets, and Transcription Factor annotation to identify and determine the functions of proteins. Mass spectrometry was outsourced to the company PTM Bio Hangzhou China.

2. Supplementary Results

2.1. ELISA Results

Each standard and 5x diluted sample was loaded in duplicate (Table 1). With the exception of wells A9 and A10 (for which the value of A9 was used directly), the average of the duplicates were taken (Table 2). This is due to a mistake in loading in A9. The value of the standard 0 pg/mL was subtracted from all other cells. Values that ended up below 0 were replaced with 0 (Table 3). The equation derived from the standard curve was BoNT/A Concentration = 39.653*O.D. (R^2= 0.9485). This was used to derive concentrations of BoNT/A for the samples loaded(Table 4). Because the samples loaded were 5x diluted, the original breastmilk BoNT/A concentrations were obtained by multiplying concentrations by 5 (Table 5).

Table 1: **Raw ELISA Measured Concentrations**.

	1	2	3	4	5	6	7	8	9	10	11	12
A	0.103	0.094	0.306	0.303	0.523	0.635	0.804	0.588	1.406	0.468	1.951	1.765
B	0.859	0.845	0.846	0.840	0.856	0.892	0.700	0.857	0.519	0.508	1.008	0.870
C	0.811	0.877	0.791	0.877	0.261	0.282	0.676	0.857	0.346	0.344	0.542	0.531
D	0.087	0.088	0.106	0.088	0.800	0.863	0.644	0.817	0.641	0.585	0.885	0.941

Table 2: **ELISA Duplicates Averaged**.

	1	2	3	4	5	6	7	8	9	10	11	12
**A**	0.0985		0.3044		0.57855		0.69595		1.406		1.765	
**B**	0.8519		0.8429		0.874		0.77805		0.5132		0.9387	
**C**	0.8439		0.834		0.27165		0.76625		0.3449		0.5364	
**D**	0.08745		0.0972		0.8317		0.7304		0.6134		0.9133	

Table 3: **ELISA Readings Zeroed**.

	1	2	3	4	5	6	7	8	9	10	11	12
**A**	0		0.2059		0.48005		0.59745		1.3075		1.6665	
**B**	0.7534		0.7444		0.7755		0.67955		0.4147		0.8402	
**C**	0.7454		0.7355		0.17315		0.66775		0.2464		0.4379	
**D**	-0.01105(0)		-0.0013 (0)		0.7332		0.6319		0.5149		0.8148	

Table 4: **Calculated BoNT/A Concentrations in (5x Diluted) Samples**.

	1	2	3	4	5	6	7	8	9	10	11	12
**A**	0 pg/mL ST0		8.19 pg/mL ST1		19.08 pg/mL ST2		23.75 pg/mL ST3		51.98 pg/mL ST4		66.248 pg/mL ST	
**B**	29.95 pg/mL W1 6 h		29.59 pg/mL W1 24 h		30.83 pg/mL W1 36 h		27.014 pg/mL W1 48 h		16.49 pg/mL W1 72 h		33.400 pg/mL W1 4 days	
**C**	29.63 pg/mL W1 5 days		29.24 pg/mL W1 7 days		6.88 pg/mL W1 11 days		26.545 pg/mL W1 2 months		9.795 pg/mL W1 4 months		17.408 pg/mL W1 5 months	
**D**	0 pg/mL no Botox		0 pg/mL no Botox		29.147 pg/mL W2 24 h		25.12 pg/mL W3 36 h		20.469 pg/mL W3 48 h		32.39 pg/mL W3 72 h	

Table 5: **Calculated BoNT/A Content in Original Samples**.

	1	2	3	4	5	6	7	8	9	10	11	12
**A**	0 pg/mL ST0		8.19 pg/mL ST1		19.08 pg/mL ST2		23.75 pg/mL ST3		51.98 pg/mL ST4		66.248 pg/mL ST	
**B**	149.75 pg/mL W1 6 h		147.95 pg/mL W1 24 h		154.15 pg/mL W1 36 h		135.07 pg/mL W1 48 h		82.45 pg/mL W1 72 h		167 pg/mL W1 4 days	
**C**	148.15 pg/mL W1 5 days		146.2 pg/mL W1 7 days		34.4 pg/mL W1 11 days		132.725 pg/mL W1 2 months		48.975 pg/mL W1 4 months		87.04 pg/mL W1 5 months	
**D**	0 pg/mL no Botox		0 pg/mL no Botox		145.735 pg/mL W2 24 h		125.6 pg/mL W3 36 h		102.345 pg/mL W3 48 h		161.95 pg/mL W3 72 h	

2.2. Western Blot Results

Western blot analyses were conducted to apply an alternative method for detection of BoNT/A proteins in the breastmilk of the three lactating women.

For the loading control, clear bands corresponding to Glyceraldehyde-3-phosphate dehydrogenase (GAPDH) were observed at around 37 kDa when 10 μL of breastmilk was analyzed, confirming that the protein concentration was sufficient and that breastmilk samples were adjusted to similar total protein content (Fig. 1(A). In the negative control lysate, as illustrated in the first wells of Figure 2 (C), samples from woman 4 and woman 5, who have never had received treatment of BoNT/A showed the absence of 150 kDa marker for BoNT/A.

The Western blots revealed the presence of fluctuating 150 kDa (Fig. 1 (B)) and 100 kDa (Fig. 1 (C)). As BoNT/A protein has a molecular weight of 150 kDa, consisting of a light chain (50 kDa) and a heavy chain (100 kDa); both chains have different functions in the neurotoxin's mechanism of action [3]. The detection utilized a primary antibody specific to the 1280 - 1292th amino acid residues of the heavy chain of the BoNT/A protein. To confirm the presence of sulfide bonds we compared between conducting reducing and non-reducing PAGE, anticipating the identification of a product approximately 150 kDa (non-reducing PAGE) or 100 kDa (reducing PAGE) in line with previous studies [4]. BoNT/A is produced as a single-chain polypeptide (150 kDa) that is inactive. Proteases nick the polypeptide chain resulting in a toxin that is pharmacologically active and consists of two chains: a heavy chain (100 kDa) and a light chain (50 kDa) connected together by a disulfide bond.

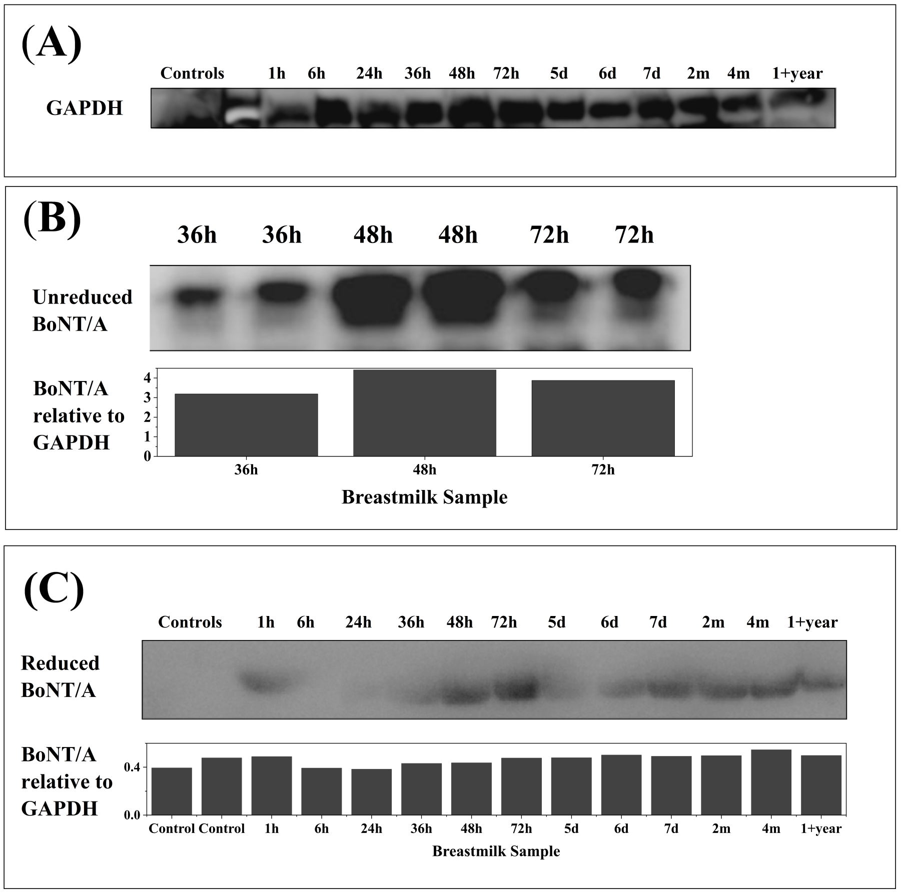

Figure 1: **Western Blot Analysis of Breastmilk Reveals Presence of BoNT/A (A)** Glyceraldehyde-3-phosphate dehydrogenase (GAPDH) loading control. GAPDH was relatively constant in all samples, indicating comparable total protein concentrations of breastmilk. **(B)** Unreduced Western Blot analysis of woman 1's breastmilk samples from 36, 48, and 72 hours. Bands around 180, 150, 120, and 45 kDa were observed. The band at around 150 kDa was most prevalent at 48 hours. Concentrated and purified woman 1 post Botox samples were run with 1 : 1000 Botulinum primary antibody, no beta mercaptoethanol, and 1 : 500 clean blot secondary antibody on a 10% gel. **(C)** Western blot analysis across a larger range of time from woman 1 was run again with an 8% gel under the same conditions. A band between at 100 kDa was observed. It was present the 1 hour, 36 hours, 48 hours, 72 hours, 5 days, 6 days, 7 days, 2 months, 4 months, and 1+ year samples but absent in the samples of women who had not received Botox before. It was also not present at 6 hours and 24 hours. This is the same molecular weight as the heavy chain of BoNT/A, between 85 - 100 kDa under reducing conditions [5]. Bands around 150 kDa and 45 kDa were also observed.

2.2.1. Unreduced Western Blot

In unreduced samples of breastmilk 36, 48, and 72 hours after injection, the highest concentration of bands at 150 kDa occurred at 48 hours (Fig. 1(A)).

2.2.2. Reduced Western Blot

Protein bands around 100 kDa are present as early as one hour post-treatment. Notably, over a longer course of time, this band peaked again on day 7 and was persistent up to over 1 year post-treatment (Fig. 1(B)).

2.3. Raman Spectroscopy Results

Next we were interested in investigating the BoNT/A concentration in breastmilk with a non-standard technique *i.e.*, confocal micro-Raman Spectroscopy. Figure 2 (A) and (B) show the Raman spectra of the samples collected from woman 1 and 2. Several characteristic peaks of breastmilk and BoNT/A were detected and summarized in Table 6. The presence of protein was identified by the amide I signature at 1656 cm^{-1} in relatively high intensity in sample 1 year compared with this band in 6 hours, 48 hours and 72 hours after Botox injection in case of the woman 1 and 2 (Fig. 2). Lipids are related to the presence of the hydrocarbon chain and can be observed in three regions: **(I)**:1200 - 1050 cm^{-1}, **(II)**: 1300 - 1250 cm^{-1} and **(III)**: 1500 - 1400 cm^{-1} (Fig. 2) [6]. Specifically, the bands in the range of 1500 - 1400 cm^{-1} and 1300 cm^{-1} are linked to scissoring and twisting vibrations of the CH_2 and CH_3 groups, respectively, while the band in the region of 1200 - 1050 cm^{-1} are related to the $C-C$ stretching vibrations [6, 7]. We observed that the peak around 1440 cm^{-1} that related to $\delta(\mathrm{CH_2})$ scissoring vibrations [6, 7] show a high-intensity magnitude in both cases, *i.e.* woman 1 and woman 2, for the 1 year after Botox sample (Fig. 2(A)). In addition, the peak at 1156 cm^{-1} that is related to $\delta\mathrm{CH_2}$ twisting vibrations [6, 7] has a relatively high-intensity magnitude in case of 48 hours and 6 hours after Botox injection samples for woman 1 and 2, respectively. Likewise, the peak at 1518 cm^{-1} which is related to $C-H$ stretching vibrations [6, 7], shows a large intensity magnitude in the case of women 1 for 48 hours and 2 for 6 hours after Botox injection, in comparison with the corresponding one Raman peak observed on the rest of the breastmilk samples. Lastly, twisting and rocking vibrations of CH_2 are noted in Raman spectrum at 870 cm^{-1} and 1156 cm^{-1} [7].

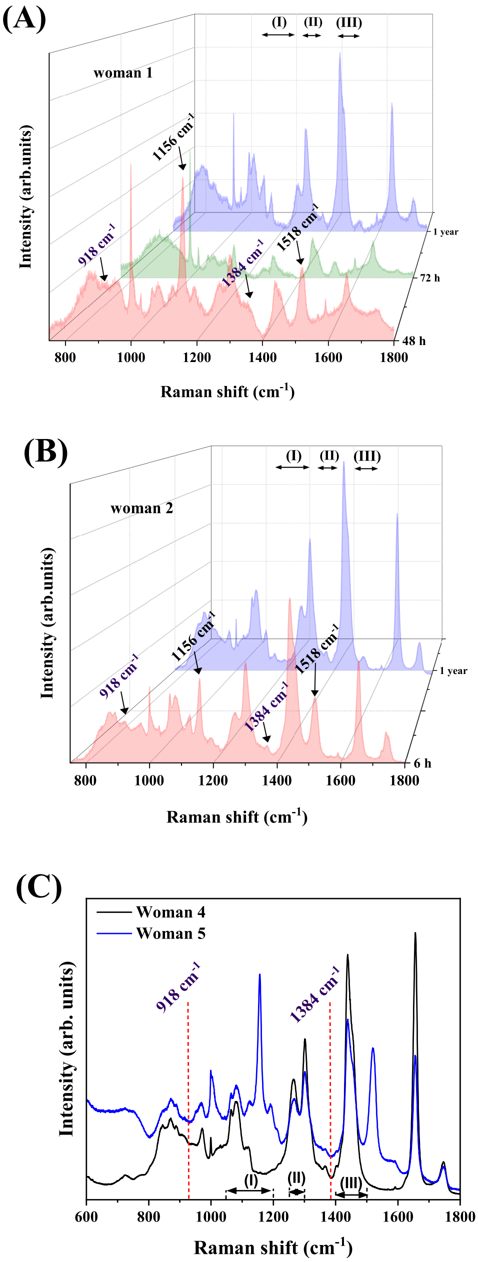

Figure 2: **Raman Spectra of Breastmilk Samples from (A) woman 1, (B) woman 2 and (C) woman 4 and 5**. The measurements were performed in a liquid environment with an excitation wavelength at 532 nm, an oil immersion objective lens with a magnification of 100×, and an integration time of 15 s. The characteristic peaks at 918 cm^{-1} and 1384 cm^{-1} were observed in samples 48 hours (woman 1) and 6 hours (woman 2) suggesting the presence of BoNT/A [8]. Note: **(I)**:1200 - 1050 cm^{-1}, **(II)**: 1300 - 1250 cm^{-1} and **(III)**: 1500 - 1400 cm^{-1}. The red dashed lines indicate the position of missing peaks in Raman spectrum of women 4 and 5.

Table 6: **Raman Peaks of Breastmilk Samples** [6, 7]

**Wavenumber** (cm^{-1})	**Vibration**
850 - 920	CH_3 rocking, CH_2 rocking
	CC backbone, C-CH_3 stretching
1000	CC symmetric stretching
1080	CC asymmetric stretching
1120 - 1123	CC anti-sym stretching
1437 - 1460	$\delta(CH_2)$ scissoring, CH_2 deformation
1656	Amide I
1749	C=O stretching

Comparing the spectra in Figures 2, we noted two Raman peaks around 918 cm^{-1} and 1384 cm^{-1} only in the samples of 48 hours (woman 1) and 6 hours (woman 2). These peaks have been detected in Clostridium Botulinum B bacterium which can produce Botox in specific conditions [8, 9].

2.4. Mass Spectrometry Results

In the 6 hours sample, 22666 peptides were detected, consisting of 22097 unique peptides and 3191 identified proteins. In the 48 hours sample, 22333 peptides were detected, with 21688 unique peptides and 3140 identified proteins. The majority of the protein fragments digested with Trypsin detected were less than 20 peptides. Therefore, most proteins were not fully covered (Fig. 3). BoNT/A was not detected among these peptides.

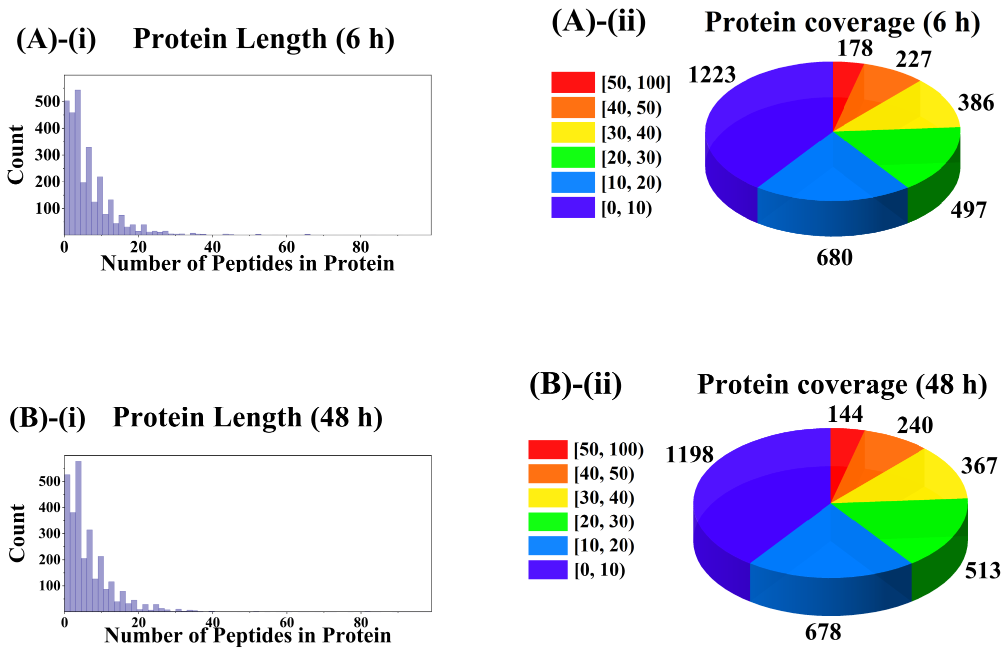

Figure 3: **Peptide Length and Protein Coverage**. Most proteins captured were less than 20 peptides long, and only a small portion of proteins were captured

3. Supplementary Discussion

Here, we present the details of the 3 non conventional methods we used for the detection and quantification of BoNT/A in breastmilk samples of three lactating women from 1 hour to over 1 year after Botox injection. These methods were Western Blot analysis, Confocal micro-Raman Spectroscopy, and Mass Spectroscopy (LC-MS).

Western blot was performed to detect BoNT/A in breastmilk samples. The specificity of the band at molecular weight 150 kDa under no reducing PAGE and 100 kDa under reducing agent beta-mercaptoethanol. This was important to confirm the presence of sulfide bonds we compared between reducing and non-reducing PAGE bonds for BoNT/A was confirmed in all lactating women who received facial injections (woman 1, 2, 3), while no presence of BoNT/A was detected in women who never received Botox treatment (women 4,5). Combined, these data indicate the potential for Western Blot as a method to detect BoNT/A. Depending on factors of antibody-antigen binding affinity and chemiluminescent substrates, femtograms, picograms, or milligrams of target protein can be detected [10]. However, because we were unable to attain Botox as run a positive control of spiked samples, our Western Blotting lacks a validated primary antibody and validated internal control. We were also unable to determine the linear range of detection for the concentrations that can be accurately quantified. These would all be future directions to validate the Western Blotting of this study as a method of BoNT/A detection.

Recent studies have shown that optical screenings and diagnoses have been widely used for characterizing biological media with reliable results including in differentiating between feeding male and female infants mother's milk [11, 12]. By utilizing confocal micro-Raman spectroscopy, we collected and analysed the Raman spectrum of breastmilk samples in order to identify BoNT/A. Two Raman peaks around 918 cm^{-1} and 1384 cm^{-1} were noted only in the samples of 48 h (woman 1) and 6 h (woman 2). This observation along with the high-intensity magnitude of Raman peaks at 1156 cm^{-1} and 1518 cm^{-1} in our Raman spectra, lead us to assume the potential presence of BoNT/A in these two samples since these two peaks have been also detected in Clostridium botulinum B bacterium [8, 9]. However, additional experiments using more sensitive techniques such as Surface-Enhanced Raman Scattering (SERS) [13] or Fano-enhanced Raman scattering (FERS) [14] could provide complementary evidence to support this observation. Similar

to the Western Blotting technique, the absence of pure Botox for calibration and the lack of spiked sample validation constrained our ability to establish a direct quantitative correlation between Raman signal intensity and Botox concentration. As a result, while our findings are supported by comparative analysis with literature-reported Raman signatures, the precise quantification of Botox in human milk samples remains unclear. Future work could benefit from acquiring pure Botox for calibration and utilizing spiked samples to enhance the accuracy and sensitivity of the assay. This would be a particularly worthwhile future direction because after the initial cost of a spectrometer, Raman spectroscopy has minimal costs, doesn't require antibodies, and can be performed in minutes.

LC-MS consists of 2 parts; Liquid chromatography (LC) is first used to separate components of a solution by mass, after which mass spectrometry (MS) is used to identify these components based on fragmentation pattern differences. It is highly sensitive, down to detection of parts per million (ppm), and can be performed quickly and efficiently [15]. However, in our results concentrations of BoNT/A were even lower than this threshold, requiring more targeted approaches.

References

[1] T. Grenda, A. Grenda, P. Krawczyk, K. Kwiatek, Botulinum toxin in cancer therapy—current perspectives and limitations, Applied Microbiology and Biotechnology 106 (2021) 485 – 495.

[2] Anti-clostridium Botulinum toxin A antibody [B364M] (ab252737), acessed on April 14, 2024 (2024).

[3] O. Rossetto, L. Morbiato, P. Caccin, M. Rigioni, C. Montecucco, Presynaptic enzymatic neurotoxins, Journal of Neurochemistry 97 (2006) 1534–1545.

[4] D. Dressler, R. Benecke, Pharmacology of therapeutic botulinum toxin preparations, Disability and Rehabilitation 29 (2007) 1761–1768.

[5] J. Weisemann, N. Krez, U. Fiebig, S. Worbs, M. Skiba, T. Endermann, M. B. Dorner, T. Bergström, A. Muñoz, I. Zegers, C. Müller, S. P. Jenkinson, M.-A. Avondet, L. Delbrassinne, S. Denayer, R. Zeleny, H. Schimmel, C. Åstot, B. G. Dorner, A. Rummel, Generation and characterization of six recombinant Botulinum neurotoxins as reference material

to serve in an international proficiency test, Toxins 7 (12) (2015) 5035–5054.

[6] K. Czamara, K. Majzner, M. Z. Pacia, K. Kochan, A. Kaczor, M. Baranska, Raman spectroscopy of lipids: A review, Journal of Raman Spectroscopy 46 (1) (2015) 4–20.

[7] E. Wiercigroch, E. Szafraniec, K. Czamara, M. Z. Pacia, K. Majzner, K. Kochan, A. Kaczor, M. Baranska, K. Malek, Raman and infrared spectroscopy of carbohydrates: A review, Spectrochimica Acta Part A: Molecular and Biomolecular Spectroscopy 185 (2017) 317–335.

[8] J. Zhang, H. Jiang, P. Gao, Y. Wu, H. Sun, Y. Huang, X. Xu, Confocal Raman microspectroscopy combined with chemometrics as a discrimination method of clostridia and serotypes of Clostridium botulinum strains, Journal of Raman Spectroscopy 52 (11) (2021) 1820–1829.

[9] M. R. M. Khorasan, M. Rahbar, A. Z. Bialvaei, M. M. Gouya, F. Shahcheraghi, B. Eshrati, Prevalence, risk factors, and epidemiology of food-borne Botulism in iran, Journal of Epidemiology and Global Health 10 (2020) 288–292.

[10] L. Pillai-Kastoori, A. R. Schutz-Geschwender, J. A. Harford, ”a systematic approach to quantitative western blot analysis”, Analytical Biochemistry (2020) 113608.

[11] E. Brauchle, K. Schenke-Layland, Raman spectroscopy in biomedicine – non-invasive in vitro analysis of cells and extracellular matrix components in tissues, Biotechnology Journal 8 (3) (2012) 288–297.

[12] R. Ullah, S. Khan, S. Javaid, H. Ali, M. Bilal, M. Saleem, Raman spectroscopy combined with a support vector machine for differentiating between feeding male and female infants mother’s milk, Biomedical Optics Express 9 (2) (2018) 844–851.

[13] N. Feliu, M. Hassan, E. Garcia Rico, D. Cui, W. Parak, R. Alvarez-Puebla, SERS quantification and characterization of proteins and other biomolecules, Langmuir 33 (38) (2017) 9711–9730.

[14] D. G. Kotsifaki, R. R. Singh, S. N. Chormaic, V. G. Truong, Asymmetric split-ring plasmonic nanostructures for the optical sensing of Escherichia coli, Biomed. Opt. Express 14 (9) (2023) 4875–4887.

[15] C. E. Doneanu, M. Anderson, B. J. Williams, M. A. Lauber, A. Chakraborty, W. Chen, Enhanced detection of low-abundance host cell protein impurities in high-purity monoclonal antibodies down to 1 ppm using ion mobility Mass Spectrometry coupled with multidimensional Liquid Chromatography, Analytical Chemistry 87 (20) (2015) 10283–10291.
